# Revisiting GNRA and UNCG folds: U-turns versus Z-turns in RNA hairpin loops

**DOI:** 10.1261/rna.059097.116

**Published:** 2017-03

**Authors:** Luigi D'Ascenzo, Filip Leonarski, Quentin Vicens, Pascal Auffinger

**Affiliations:** 1 Université de Strasbourg, CNRS, Architecture et Réactivité de l'ARN, UPR 9002, F-67000 Strasbourg, France; 2 Faculty of Chemistry, University of Warsaw, 02-093 Warsaw, Poland

**Keywords:** RNA folding, RNA motif, structure prediction, tetraloop, tRNA anticodon

## Abstract

When thinking about RNA three-dimensional structures, coming across GNRA and UNCG tetraloops is perceived as a boon since their folds have been extensively described. Nevertheless, analyzing loop conformations within RNA and RNP structures led us to uncover several instances of GNRA and UNCG loops that do not fold as expected. We noticed that when a GNRA does not assume its “natural” fold, it adopts the one we typically associate with a UNCG sequence. The same folding interconversion may occur for loops with UNCG sequences, for instance within tRNA anticodon loops. Hence, we show that some structured tetranucleotide sequences starting with G or U can adopt either of these folds. The underlying structural basis that defines these two fold types is the mutually exclusive stacking of a backbone oxygen on either the first (in GNRA) or the last nucleobase (in UNCG), generating an oxygen–π contact. We thereby propose to refrain from using sequences to distinguish between loop conformations. Instead, we suggest using descriptors such as U-turn (for “GNRA-type” folds) and a newly described Z-turn (for “UNCG-type” folds). Because tetraloops adopt for the largest part only two (inter)convertible turns, we are better able to interpret from a structural perspective loop interchangeability occurring in ribosomes and viral RNA. In this respect, we propose a general view on the inclination for a given sequence to adopt (or not) a specific fold. We also suggest how long-noncoding RNAs may adopt discrete but transient structures, which are therefore hard to predict.

## INTRODUCTION

RNA architecture is modular and hierarchical, which implies that secondary structural elements such as double stranded helices, hairpins, and single-stranded loops are linked by tertiary interactions that guide the assembly process (Hendrix et al. 2005; Cruz and Westhof 2009; Butcher and Pyle 2011). The majority of hairpin stems are capped by GNRA or UNCG tetranucleotide sequences—where N is any base and R is a purine (Cheong et al. 2015; Hall 2015). These tetranucleotide loops adopt distinctive folds that involve extensive and well-described networks of hydrogen bonds and stacking interactions (Cheong et al. 1990; Heus and Pardi 1991; Allain and Varani 1995; Jucker and Pardi 1995a; Jucker et al. 1996; Ennifar et al. 2000; Correll and Swinger 2003; Nozinovic et al. 2010). For GNRA and UNCG loops, it is generally assumed that the sequence commands a unique fold. Hence, upon considering sequence alignments and secondary structures of RNA families for which no 3D structures are available, we presume that we understand how these tetraloops fold.

Here, we present structural evidence that challenges these expectations by identifying GNRA sequences that adopt a UNCG fold and vice versa, both in tetraloops closed by a Watson–Crick base pair and in tetraloop-like motifs embedded in larger ribosomal and tRNA loops (Auffinger and Westhof 2001). Although this loop dimorphism remains rare within the pool of RNAs for which we currently possess 3D data, it led us to question some basic assumptions we make about RNA folding and structure prediction.

To better characterize these interconversions, we propose a more general structure-based tetraloop and tetraloop-like identification scheme that involves on one side the classical and well-described U-turn (Gutell et al. 2000) and, on the other, a newly defined “Z-turn,” which is based on the UNCG tetraloop fold and the Z-RNA CpG step it encompasses (D'Ascenzo et al. 2016). We establish that these two turns and variants thereof are key to the tetraloop and tetraloop-like folding landscape, but also to most turns in RNAs. A typical and infrequent tetranucleotide fold that does not conform to these rules will be described in more detail elsewhere. Here, before pursuing, we need first to (re)define U-turns and Z-turns as they appear in structured tetranucleotide folds within hairpins (see also Materials and Methods).

### U-turn and U_SH_-turn signatures

**FIGURE 1.**
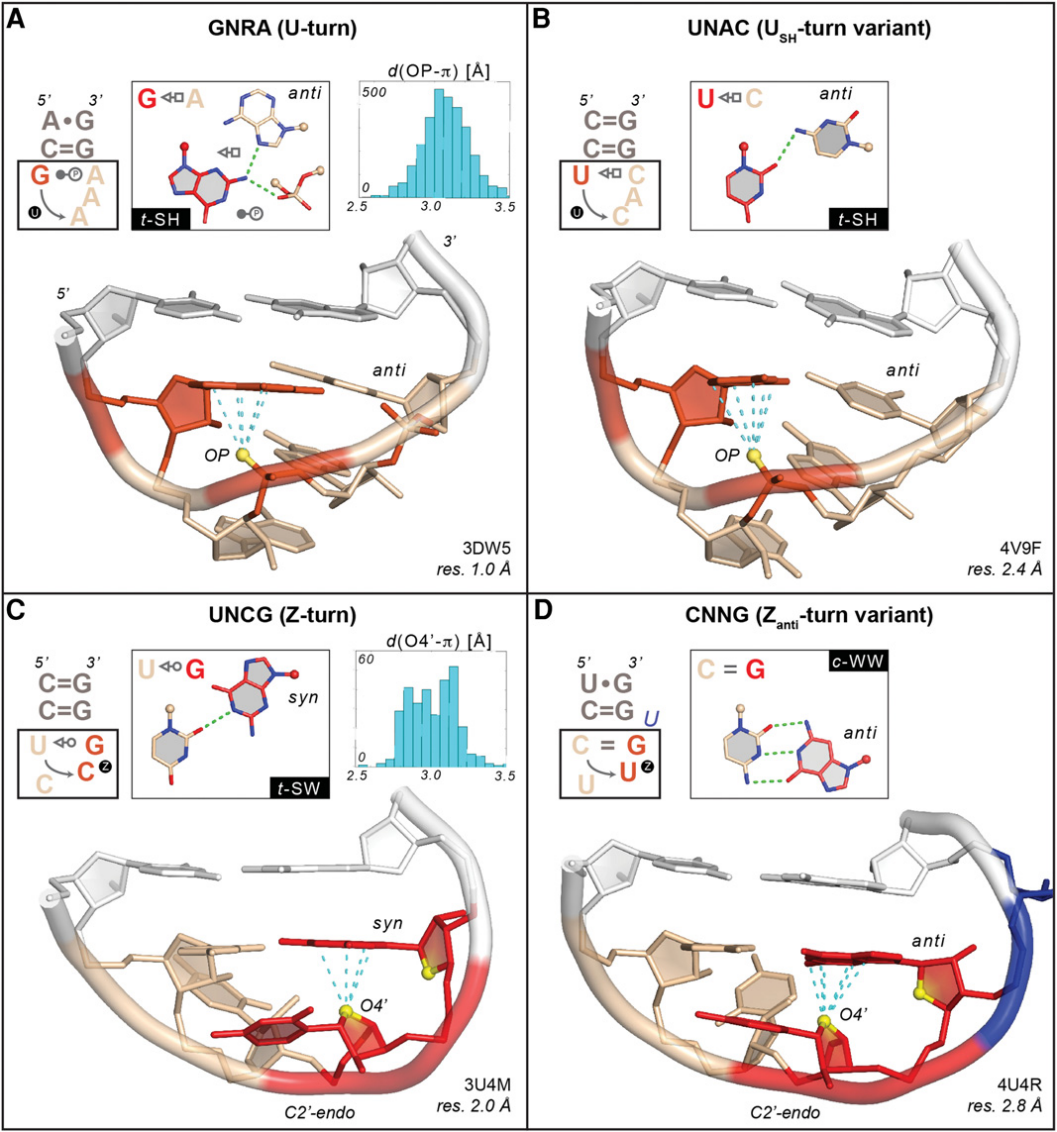
Examples of a GNRA “U-turn” and a UNCG “Z-turn” along with their U_SH_-turn and Z_anti_-turn variants (1–4 bp and relevant nucleobase–phosphate hydrogen bonds are shown in the *insets*). In all panels, the cyan dashed lines mark contact distances between the OP/O4′ atoms—emphasized as yellow spheres—and the stacked nucleobase that are associated with oxygen-π contacts ≤3.5 Å (see Materials and Methods and *insets* of panels *A* and *C*). For clarity, all nonrelevant OP atoms were hidden. The C = G closing base pairs are shown in white. For all secondary structures, symbols according to the Leontis and Westhof nomenclature were used (Leontis and Westhof 2001; Nasalean et al. 2009). (*A*) G_2659_AAA tetraloop (chain A) adopting a classical U-turn (symbolized by a circled “U”). The first G and the phosphate of the third nucleotide involved in an OP–π contact are marked in red as well as the oxygen atoms of the phosphate involved in the 1–4 base–phosphate hydrogen bond. The three stacked A nucleotides are colored in wheat. (*B*) U_253_CAC tetraloop (chain 0) adopting the rare U_SH_-turn variant (symbolized by a circled “U”). The first U and the phosphate of the third nucleotide are marked in red. The three stacked CAC nucleobases and part of their backbone are colored in wheat. (*C*) U_2144_CCG tetraloop (chain B) adopting a Z-turn (symbolized by a circled “Z”). The CpG step forming a Z-RNA motif is shown in red. The two ribose O4′ atoms of the CpG step are shown in yellow to mark the characteristic head-to-tail orientation of the sugars. The fourth nucleotide adopts a *syn* conformation. The UpC step is colored in wheat. (*D*) C_3194_UUGu pentaloop (chain 1) adopting a rare Z_anti_-turn variant (symbolized by a circled “Z”). The UpG step forming a Z-RNA motif, with the G adopting an *anti* instead of a *syn* conformation, is shown in red. The two ribose O4′ atoms of the CpG step are shown in yellow to mark the characteristic head-to-tail orientation of the sugars. The CpU step is colored in wheat and the bulged “u” in blue.

A U-turn is a tetranucleotide motif that was first identified in tRNA anticodon and T-loops (Quigley and Rich 1976; Gutell et al. 2000; Auffinger and Westhof 2001; Klosterman et al. 2004) and has since been characterized in a large variety of structural motifs starting with a uridine or a pseudouridine. In that respect, U-turns were sometimes called uridine-turns or π-turns (Kim and Sussman 1976; Jucker and Pardi 1995a). U-turns were also associated with “G-starting” motifs such as GNRA tetraloops (Fig. 1A), or more recently in tetranucleotide motifs involving a protonated cytosine like a uC^+^UAAu loop (Gottstein-Schmidtke et al. 2014). In short, a U-turn involves a hydrogen bond between the first nucleobase—with a U/G/C^+^ imino or amino nitrogen atom—and an OP atom of the fourth nucleotide. This base–phosphate hydrogen bond is of the “5/4/3BPh” type according to a recent classification (Zirbel et al. 2009). It ensues that the 1–4 G•A *trans*-Sugar/Watson–Crick pair (*t*–SW) occurring in GNRA loops should not be considered as a U-turn determinant although it is essential for interactions with GNRA receptors (Fiore and Nesbitt 2013).

As an important outcome, the characteristic 1–4 nucleobase–phosphate (or nucleobase–OP) hydrogen bond imposes the formation of an oxygen–π or phosphate–π stacking contact between the first nucleobase and an OP atom of the third nucleotide. A PDB survey led to an average OP–π stacking distance of 3.0 ± 0.2 Å, with a maximum distance of 3.5 Å. This oxygen–π contact, which is a further characteristic of U-turns, has rarely been described (Egli and Sarkhel 2007).

It emerges that these two features, namely the 1–4 nucleobase–OP hydrogen bond and the OP–π stacking contacts, are sufficient to unambiguously characterize a U-turn. The latter criterion allows us also to distinguish between regular and partially degenerated or unfolded U-turns, which correspond to loops with no oxygen–π stacking contact and are most often found at RNA–protein interfaces. However, such occurrences are rare (see the following section).

A U-turn variant has been identified for UNAC sequences (Fig. 1B). These loops were found to mimic GNRA tetraloops since their backbone conformations are similar (Zhao et al. 2012). The 1–4 interaction involves a U•C *trans*-Sugar/Hoogsteen (*t*-SH) pair instead of a hydrogen bond involving the OP atom of the fourth nucleotide as in more typical U-turns. Yet, in the examples we collected, the OP–π contact between the first nucleobase and an OP atom of the third nucleotide is conserved. In the following, we call this U-turn variant a “U_SH_-turn” because of the consistent presence of a 1–4 *t*–SH pair.

Note that the cGANCg tetraloop in group IIC introns has a backbone that is similar to that of a U-turn and a 1–4 G•A *t*–SW pair (Keating et al. 2008). Although rare, these GANC loops are examples of structured tetraloops with no oxygen–π contact. For all U-turns, it is important to note that the last three nucleobases are stacked in a manner that their exposed Watson–Crick edges can establish specific tertiary contacts such as, for example, within anticodon–codon associations or with cognate receptors (Fiore and Nesbitt 2013; Tanaka et al. 2013).

### Z-turn and Z_anti_-turn signatures

UNCG tetraloops are not based on a U-turn but on a newly defined “Z-turn”: they embed a *trans*-Sugar/Watson–Crick (*t*–SW) interaction between the first and fourth nucleobase, associated with a *C2′-endo* pucker of the third residue, and a *syn* conformation of the fourth residue. In addition, the third and fourth ribose rings adopt an uncommon head-to-tail orientation (Fig. 1C). This particular combination of rare structural features is characteristic of Z–DNA/RNA motifs and implies an O4′–π stacking contact (Egli and Sarkhel 2007; D'Ascenzo et al. 2016). The 3–4 O4′–π stacking contact in Z-turns is comparable with the 1–3 OP–π stacking contact in U-turns. Furthermore, the average stacking distance (3.1 ± 0.2 Å) and the maximum distance (3.5 Å) are similar in both turns. Thus, we can assume that to define a Z-turn as found in UNCG loops, we can rely on both the 1–4 base pair essentially of the *t*–SW type as described below, and the O4′–π stacking contact.

Such a definition is not based on the *syn* conformation of the fourth nucleotide and therefore allows us to consider rare motifs where the O4′ stacking involves bases in *anti*, such as found in some CUUG folds (Fig. 1D; Jucker and Pardi 1995b). Hence, as for U-turns, we can define two Z-turn subcategories: the main Z-turn or Z_syn_-turn—with the fourth nucleobase in *syn*—and the less frequent “Z_anti_-turn” variant—with the fourth nucleobase in *anti*. Most Z_anti_-turns are not associated with a *t*–SW 1–4 pair but with a *cis*-Watson–Crick/Watson–Crick (*c*-WW) pair. As such, these Z_anti_-turns are also known as di-loops. Interestingly, the characteristic *C2′-endo* sugar pucker of UNCG tetraloops seems to be conserved in all Z-turn types.

### U-turns and Z-turns dominate the tetranucleotide folding landscape in RNA hairpins

In our unified definition of U-turns and Z-turns in RNA hairpins, each turn is distinguished by the presence of either a 1–3 or 3–4 oxygen–π contact (Egli and Sarkhel 2007). With the above-defined criteria, we searched the PDB for occurrences of these two turns and their variants in crystal and NMR structures, among tetranucleotide sequences embedded in RNA hairpin loops (Table 1). As expected, U-turns in tetranucleotide sequences starting with G, U, or C^+^ are the most frequent, followed by Z-turns in UNCG tetraloops. U_SH_-turns are less frequent and are associated with UNAC sequences. Z_anti_-turns are slightly more frequent and diverse and comprise essentially CNNG sequences. The “Uncategorized” motifs are mostly of the partially unfolded U-turn type—where the 1–4 interaction is present, but not the OP–π stacking contact. They correspond also to folds that are too rare and/or disordered to allow for their assignment to any clearly defined category, or to partially unfolded conformations induced by proteins. The rare GANC tetranucleotide loop has only been identified in group IIC introns based on structural and phylogenetic evidence and has only been reported when bound to its cognate receptor (Keating et al. 2008). Thus, our early assumption that the largest part of tetranucleotide folds in hairpins is based on a U-turn or a Z-turn comprising an oxygen–π stacking contact is supported by this survey. Consequently, we can assume that most GNRA and UNCG tetranucleotide fold predictions based on sequence alignments are correct (Table 1).

**TABLE 1.**
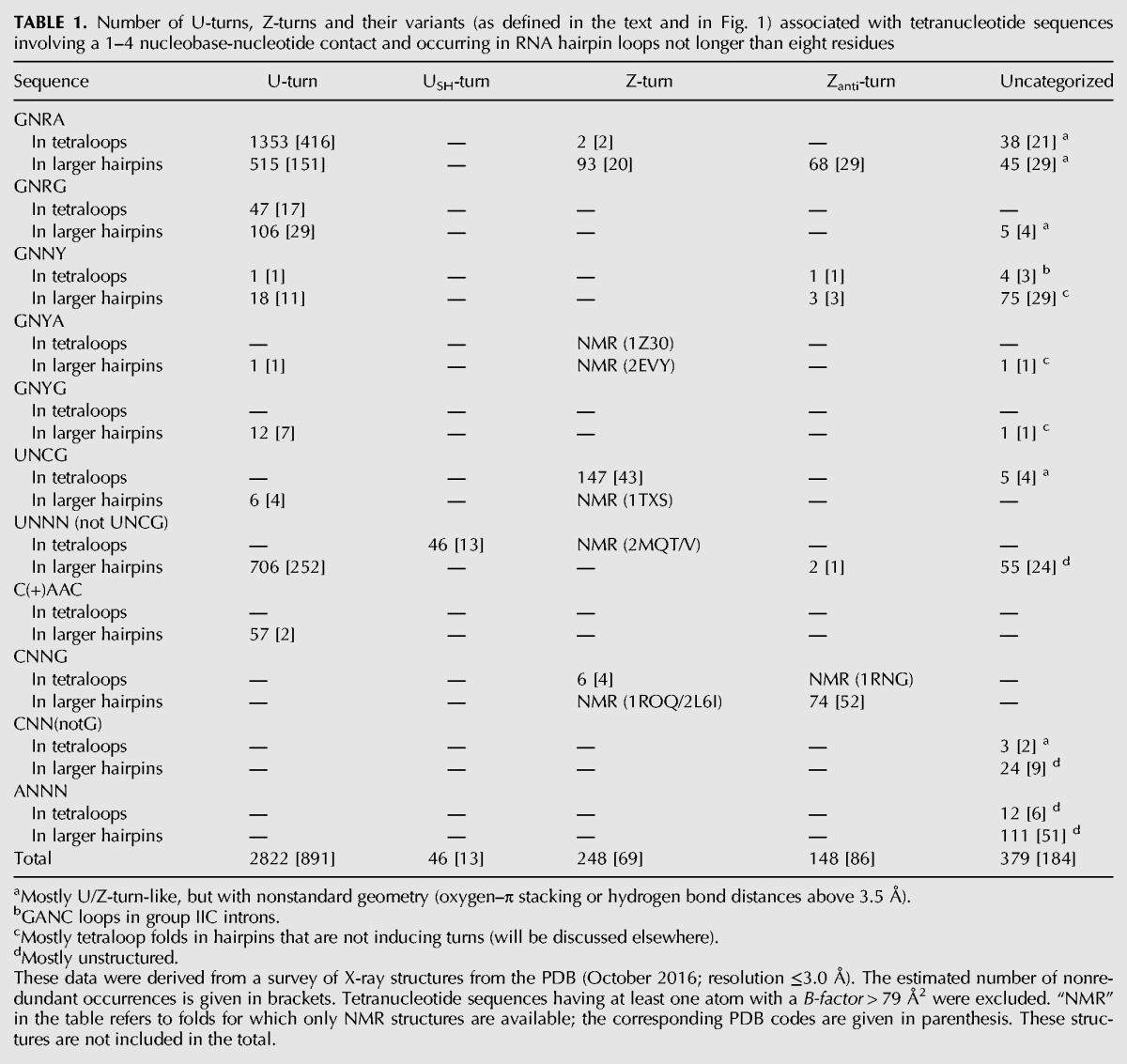
Number of U-turns, Z-turns and their variants (as defined in the text and in Fig. 1) associated with tetranucleotide sequences involving a 1–4 nucleobase-nucleotide contact and occurring in RNA hairpin loops not longer than eight residues

**FIGURE 2.**
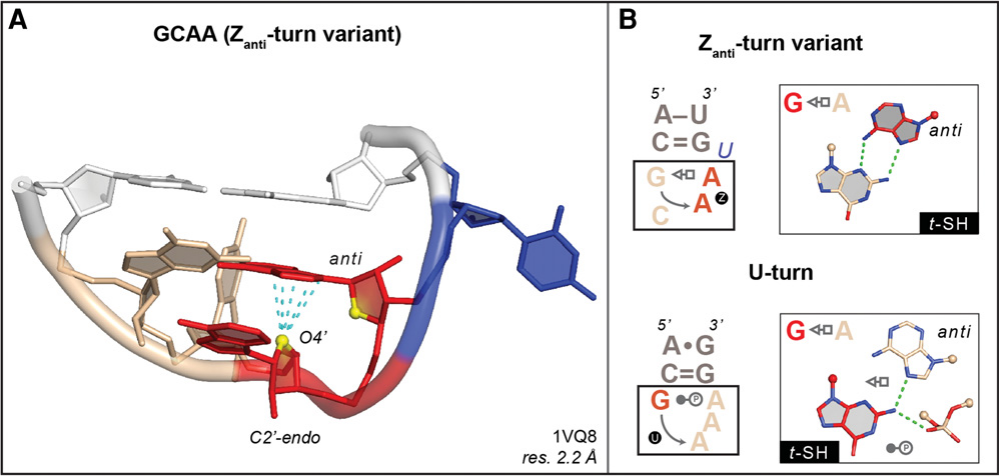
G_196_CAAu sequence (chain 0) adopting a rare Z_anti_-turn variant. (*A*) The ApA step forming a Z-RNA motif, with the A adopting an *anti* instead of a *syn* conformation, is shown in red. The two ribose O4′ atoms of the CpG step are shown in yellow to mark the characteristic head-to-tail orientation of the sugars. The GpC step is colored in wheat, the bulged U in blue, and the closing base pair in white. (*B*) Comparison of the secondary structures and of the associated 1–4 G•A *t*-SH pairs for the Z_anti_- and the U-turns, to emphasize their differences. See also Figure 1A for the GAAA U-turn.

**FIGURE 3.**
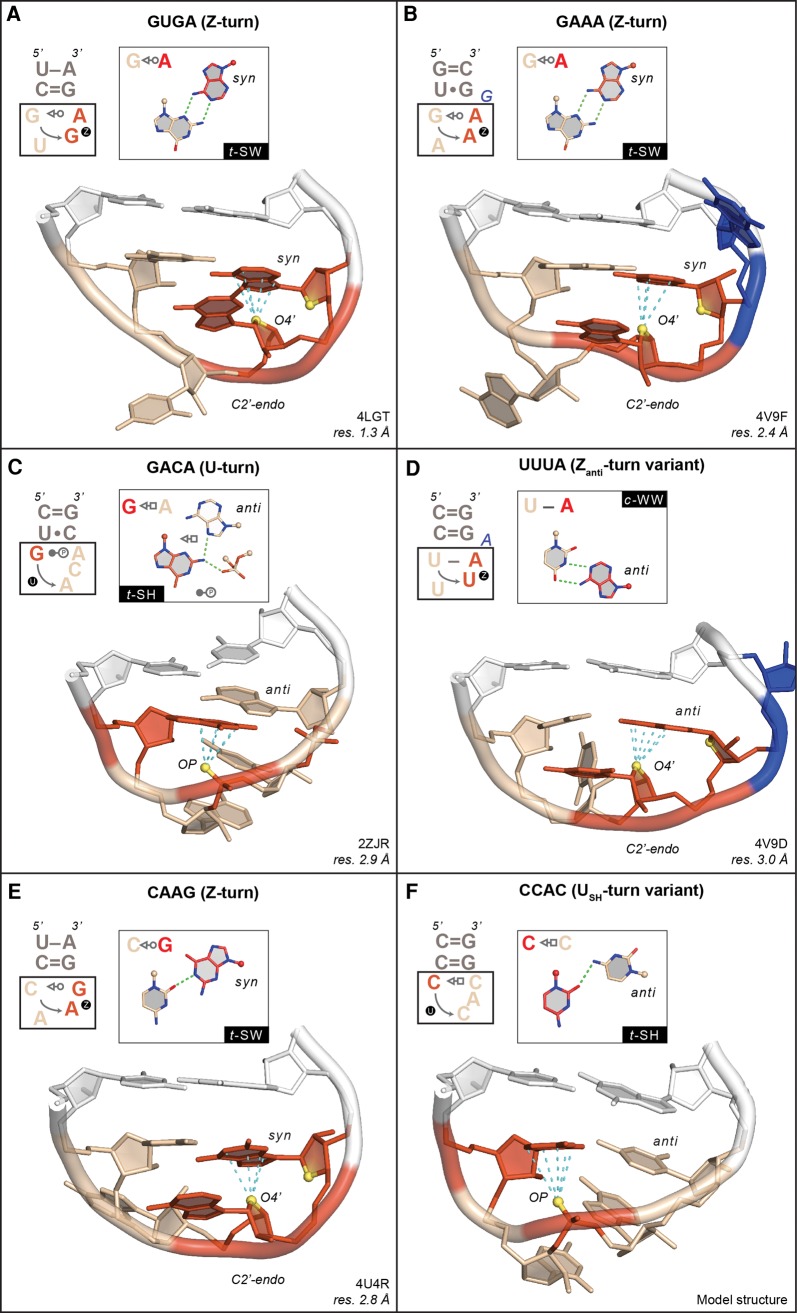
Examples of tetranucleotide sequences adopting unanticipated folds (1–4 bp are shown in the *insets*). In all panels, the cyan dashed lines mark contact distances between the OP/O4′ atoms—in yellow—and the stacked nucleobase that are associated with oxygen–π contacts ≤3.5 Å (see Materials and Methods). For clarity, all nonessential OP atoms were hidden. All closing base pairs are shown in white. All turns are symbolized by a circled “U” or “Z” as in Figure 1. (*A*) G_2595_UGA sequence (chain E) adopting a Z-turn. The Z-RNA GpA step is shown in red. The O4′ atoms of the two GpA riboses are shown in yellow to mark the characteristic head-to-tail orientation of the sugars. The GpU step is colored in wheat. (*B*) G_873_AAAg sequence (chain 0) embedded in a 7-nt loop and adopting a Z-turn. The ApA step that forms a Z-RNA motif is shown in red. The O4′ atoms of the two ApA riboses are shown in yellow to mark the characteristic head-to-tail orientation of the sugars. The GpA step is colored in wheat; the bulged “g” nucleotide is shown in blue. (*C*) G_2796_ACA sequence (chain X) adopting a classical U-turn. The first G and the phosphate of the third nucleotide involved in an OP–π contact are marked in red as well as the oxygen atoms of the phosphate involved in the 1–4 base–phosphate contact. The stacked ACA nucleotides are colored in wheat. (*D*) U_2595_UUAa sequence (chain DA) adopting a Z_anti_-turn. The UpA step that forms a Z-RNA motif is shown in red. The O4′ atoms of the two UpA riboses are shown in yellow to mark the characteristic head-to-tail orientation of the sugars. The UpU step is colored in wheat; the bulged “a” nucleotide is shown in blue. (*E*) C_415_AAG sequence (chain 2) adopting a Z-turn. The ApG step that forms a Z-RNA motif is shown in red. The O4′ atoms of the two ApG riboses are shown in yellow to mark the characteristic head-to-tail orientation of the sugars. The CpA step is colored in wheat. (*F*) Model structure of a CCAC sequence adopting a U_SH_-turn. The first C and the phosphate of the third nucleotide involved in an OP–π contact are marked in red. The three stacked CAC nucleotides are colored in wheat.

However, these data also indicate that some sequences expected to form a U-turn are associated with a Z-turn and vice versa. Thus, the sequence of a tetraloop does not systematically dictate its fold. For instance, we identified a GCAAu sequence that adopts a Z_anti_-turn (Fig. 2). Further, one GUGA sequence of the GNRA type adopting a Z-turn was observed in an RNA–protein complex (Fig. 3A). NMR structures of anticodon loops containing the U_33_NCG sequence were found to adopt a Z-turn under specific conditions, in agreement with their sequence but not with the expected anticodon–codon binding scheme (see below). These examples are more thoroughly described in the following sections. A detailed report describing the structural features of tetranucleotide folds will be provided elsewhere, the main purpose of this account being to establish the interchangeability between U-turns and Z-turns.

### GNRA and GNYA dimorphism

Loop dimorphism came upon us serendipitously. We found that it deserved special attention, as we realized that it impacted our ability to derive three-dimensional structures from secondary structures. Upon looking at GNRA and GNYA loops, we noted that the phylogenetically conserved cGUGAg loop that caps helix 93 in domain V of all large ribosomal subunits adopts the expected U-turn. However, the same cGUGAg loop located within a 21-nt-long ribosomal fragment in a complex with a pseudouridine synthase adopts an unexpected Z-turn, which is made possible through the formation of a 1–4 G•A *t*–SW pair (Fig. 3A; Czudnochowski et al. 2014). Whether the Z-turn is induced by the pseudouridine synthase or by crystal constraints is unclear. However, it is tempting to speculate that some RNA binding proteins and modification enzymes could recognize and/or induce Z-turns in GNRA sequences.

Loop dimorphism was also observed in larger motifs containing GNRA sequences, such as the phylogenetically conserved 7-nt uGAAAgg loop that caps helix 35a in domain II of large ribosomal subunits (Hsiao et al. 2006; Nasalean et al. 2009; D'Ascenzo et al. 2016). In every X-ray and cryo-EM structure of a ribosome available to date (including mitochondrial ribosomes), this uGAAAgg—or uGACAgg in *Homo sapiens* mitochondrial ribosomes (PDB code: 4WT8; resolution: 3.4 Å) (Amunts et al. 2015)—adopts a Z-turn (Fig. 3B). Although it is imaginable that this GAAA sequence would not be folding like a regular GAAA tetraloop due to the larger size of the loop, we would probably have had difficulties in anticipating its Z-turn fold. However, to us, the most surprising example of a GNRA Z-turn—more precisely a Z_anti_-turn—is a GCAAu pentaloop observed in X-ray structures of *Haloarcula marismortui* large subunits where it caps helix 12 within domain I. This GCAA Z_anti_-turn shares a 1–4 *t*-SH G•A pair with a GNRA U-turn (see Figs. 1A, 2).

Further evidence of an exchange between U-turns and Z-turns originates from a combination of crystallographic and NMR data, which revealed that GNYA tetraloops—where Y is any pyrimidine—could fold like GNRA and adopt a U-turn since they can potentially form a 1–4 G•A *t*-SH pair (Melchers et al. 2006). However, such loops are rare in X-ray structures. Up to now, besides the uGACAg located in the above-mentioned 4WT8 cryo-EM *Homo sapiens* mitochondrial ribosome, only one X-ray occurrence of a uGACAc in *Deinoccocus radiodurans* (Fig. 3C) has been reported, where the tetranucleotide sequence adopts a U-turn (Table 1). Yet, NMR experiments illustrated that a cGUUAg loop (Ihle et al. 2005) and a uGCUAg loop (Melchers et al. 2006) can adopt a Z-turn rather than the anticipated U-turn (PDB codes: 1Z30 and 2EVY).

Overall, although such dimorphism is not frequent among structured RNAs (Table 1), it might be relevant when deriving the structures of noncoding RNA that may adopt several transient folds in order to achieve their functions within a large diversity of environments (Cech and Steitz 2014). It would therefore be interesting to explore how such conformational changes occur in vivo, especially since an *anti* to *syn* conversion could not easily be fathomed without stem unwinding.

### UNCG dimorphism: U-turns or Z-turns in tRNA anticodon loops?

It is generally well appreciated that longer loops—from pentaloops to larger motifs—can embed tetranucleotide sequences that adopt U-turns (Hsiao et al. 2006). One of the most biologically relevant systems to incorporate this fold is the 7-nt-long tRNA anticodon loop. In the context of protein synthesis, any U_33_NNN sequence will adopt a U-turn (Auffinger and Westhof 2001) so that the three anticodon bases are able to associate with the three complementary bases of the codon on the messenger RNA (mRNA). However, would a U_33_NCG anticodon sequence naturally adopt that classical U-turn conformation required for translation instead of the more cogent Z-turn? Do such anticodon loops manage to switch from U-turns to Z-turns and, if yes, which environmental context would direct such a structural transition or impose one over the other fold?

In that respect, it could be envisaged that nucleotide modifications play a role in facilitating or preventing U_33_NCG anticodon loops from adopting a Z-turn. NMR experiments were performed on four variants of tRNA^Arg1,2^ stem–loops possessing a U_33_ACG sequence and containing diverse combinations of RNA modifications such as A_34_/I and C_32_/S^2^C—PDB codes: 2KRP/Q/V/W (Cantara et al. 2012). This study revealed that all modified and nonmodified anticodon loops adopt a Z-turn, although the absence of a natural m^2^A_37_ post-transcriptional modification could have biased the outcome. In any case, it seems fair to state that the extent of nucleotide modifications modulates the conformational plasticity of the tRNA^Arg1,2^ anticodon loop in order to secure the essential U-turn conformation (Sundaram et al. 2000). However, in its unmodified state, the loop could also adopt a Z-turn and be recognized by specific proteins, as in the above-mentioned 4LGT pseudouridine synthase complex (Fig. 3A).

To summarize, these U_33_ACG anticodon sequences can successively adopt at least three distinct folds. They journey from a Z-turn in their free state, through a “degenerated” fold when bound to their cognate tRNA synthetases—see for example, tRNA^Arg^ with a U_33_ICG anticodon; PDB code: 1F7U (Delagoutte et al. 2000)—to end with a classical U-turn when interacting with mRNA codons. RNA modifications—or their absence—may determine how anticodon loops fold, thereby altering or suppressing the tRNA codon-reading capacity.

Could Z-turns of U_33_NCG anticodon loop sequences be associated with a specific biological function? Would a Z-turn be necessary for the recognition of modification sites by tRNA synthases? In that case, could Z-turns within anticodon loops also occur when other NpG steps replace CpG within the U_33_NCG sequence? After all, it has been established that almost all dinucleotide sequences can adopt Z–RNA conformations (see Fig. 3A,B for GpA and ApA Z-steps) and therefore be part of Z-turns (D'Ascenzo et al. 2016). Indeed, a NMR structure of a UCAGu pentaloop with an ApG Z-step has been reported—PDB code: 1Q75 (Theimer et al. 2003). If that hypothesis holds true, 16 out of the 64 anticodon sequences ending with a G—thereby comprising the four U_33_NCG sequences—could potentially adopt a Z-turn. Our understanding of translation regulation, of decoding rules and of the role of modified bases in tRNAs could be expanded by these findings (Grosjean and Westhof 2016).

Are other folds possible for U_33_NNN sequences? A different UGAA fold has been reported in the NMR structure of an RNA hairpin—PDB code: 1AFX (Butcher et al. 1997). However, we did not consider this fold since no 1–4 interaction was present and since this loop has not been reported elsewhere. We already described UNAC sequences (Zhao et al. 2012) that can adopt the alternative U_SH_-turn variant, where the fold is made possible by the presence of a C36 nucleotide forming a 1–4 U•C *t*–SH pair (Fig. 1B). We also identified a UUUAa pentanucleotide sequence in a ribosome structure that adopts the Z_anti_-turn variant and that is closed by a 1–4 U–A *c*-WW pair (Fig. 3D). Thus, U_33_NNN anticodon loops can theoretically adopt any of the four folds we described, depending on the nature of nucleotide 36 and the associated structural context. Although most of these folds are rarely found in experimental structures, they can transiently appear in the folding pathways of these loops depending on sequence and modification levels.

### Which turns for CNNN and ANNN sequences?

Similarly, we wondered whether CNNN sequences adopt a unique fold specific to their sequence or multiple conformations. When the C nucleotide is protonated, typical U-turns can be formed as shown by NMR and in ribosomes—see C_1469_AACu in *Haloarcula marismortui* (Gottstein-Schmidtke et al. 2014). It was inferred from NMR and thermodynamic measurements (Proctor et al. 2002) as well as X-ray crystallography (Fig. 3E) that CNNG sequences can form either Z-turns—PDB code: 1ROQ—(Du et al. 2003; Oberstrass et al. 2006; Schwalbe et al. 2008), or Z_anti_-turns. For the latter, the 1–4 C = G *c*-WW pair was significantly buckled, probably due to constraints imposed by the “di-loop” fold—PDB code: 1RNG (Jucker and Pardi 1995b). Interestingly, the cCAAGg loop that caps helix 14 of the small subunits of eukaryotic ribosomes (Fig. 3E) takes the place of a UACG loop in bacterial ribosomes, both forming a Z-turn. Besides UNNC, CNNC sequences could potentially form U_SH_-turns, although the latter have not yet been observed (Fig. 3F). Again, these loops starting with a C residue display an unanticipated plasticity, suggesting that the fold they adopt is largely context dependent.

Tetranucleotide sequences starting with an adenine are almost nonexistent, at least in crystallographic structures (Table 1). If they exist, they do not seem to display a significant and/or stable 1–4 contact as reported for the other loops described here. Hence, especially when the loop interacts with a protein, it is difficult to refer to these tetranucleotides as being “structured.” However, we do not exclude the possibility that additional motifs might emerge in newly deposited crystal or NMR structures. For instance, since a UUUAa pentaloop with a Z_anti_-turn implying a 1–4 U–A *c*-WW pair was observed, an ANNUn pentaloop with a similar turn and a 1–4 A–U pair cannot be dismissed. Such possibilities have been reported by NMR for uGUUC and CUUGu pentaloops adopting Z_anti_-turns with a 1–4 G = C or C = G *c*-WW pair—PDB code: 2L6I (Lee et al. 2011).

### Phylogenetic considerations on tetranucleotide loops in RNA

Phylogenetic data on 16S rRNA suggested early on that helix 6 (positions 83–86 in *Escherichia coli* 16S rRNA) is capped either by a CUUG (45%), a UUCG (36%), or a GCAA (13%) tetraloop (Woese et al. 1990; Konings and Gutell 1995). Thus, it could be concluded that this stem can be capped either by a Z–turn or by a U-turn. According to our present study, these three sequences can also adopt a Z-turn. Such loop polymorphism might complicate the interpretation of biochemical data, for example, when highly conserved GAAA tetraloops in 16S rRNA are substituted by a UACG sequence (Sahu et al. 2012). In addition, the fact that this loop is unstructured in the 4YBB *Escherichia coli* crystal structure (resolution: 2.1 Å) might interrogate classical phylogenetic data interpretations. Indeed, in the seven UNCG tetranucleotide sequences deduced from the 16S *Escherichia coli* 2D structure, only three adopt a canonical Z-turn and the other sequences appear in disordered regions with, however, a G nucleotide in *syn* for four of them. The reasons as to why these loops appear as disordered are not yet understood.

Thus, sequence interchangeability might be hiding structural similarity. As noted above, the Z-turn GAAA loop capping helix 35a in the 50S of *Haloarcula marismortui* could exchange with YNMG sequences. Further, convincing evidence of sequence exchange that leads to similar folds has been reported in studies of viral RNA hairpins (Melchers et al. 2006; Liu et al. 2009; Zoll et al. 2011; Clabbers et al. 2014; Prostova et al. 2015).

### Sequence–structure relationships

**FIGURE 4.**
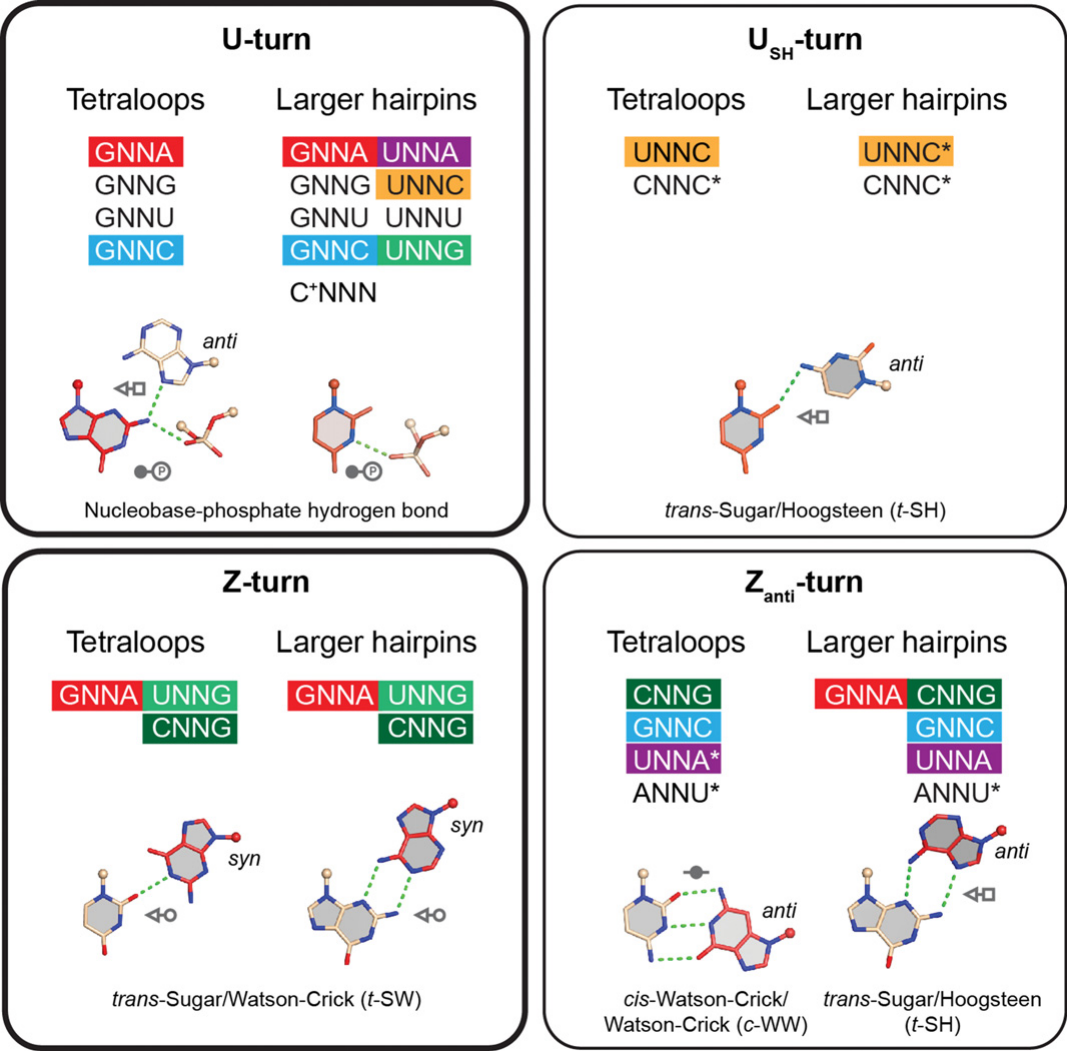
Graphical representation of the sequence–structure relationships for the four—two main and two minor—tetranucleotide turns that we characterized in RNA hairpins. The nucleobase in red is associated with a 1–3 or 3–4 oxygen–π stacking contact. The folds associated with sequences marked by an asterisk are theoretically possible but have not yet been observed in experimental structures. Here, we consider only the first and fourth nucleotides. Sequence–structure relationships associated with the second and third nucleotides will be discussed elsewhere.

It is our hope that the data we gathered (summarized in Fig. 4) will help to interpret tetranucleotide sequence variations from a structural perspective, as they inform on the prevalence of a sequence to adopt (or not) a given fold. For example, GNNA sequences with a 1–4 G•A base pair can adopt a classical GNRA U-turn fold but also a Z-turn and even a Z_anti_-turn, but not a U_SH_-turn. Similarly, UNNG sequences can adopt U-turns and Z-turns, but not the two other less frequent variants. Finally, the GNNG and GNNU sequences are only found in the U-turn category. This classification reflects our current understanding of tetranucleotide turns and might be completed or refined with the advent of new noncoding RNA structures.

### Final thoughts about folds and structure prediction

We report that tetraloop and tetranucleotide folds are not systematically determined by their sequence, possibly because of subtle changes in their environment and in the sequence of connected residues. A logical implication of this observation is that, for any given RNA sequence for which the 3D structure is not available, we are unable to ascertain with 100% confidence how the hairpins it contains will fold. With prior knowledge acquired on ribozymes (Schultes and Bartel 2000; Woodson 2015) and riboswitches (Garst et al. 2011; Batey 2015), we became aware that the same RNA sequence can adopt distinct folds in order to carry out specific functions. The structural analysis we present here reveals that only two folds dominate the tetranucleotide landscape. Consequently, predicting whether GNRA, UNCG, or related sequences within any noncoding RNA will adopt a U-turn involving a phosphate–π stacking contact or a Z-turn with a O4′–π stacking ceases to be a straightforward exercise. Without additional stereochemical rules, the structure adopted by such tetranucleotide sequences might remain complex to predict and more structural information on these essential folds needs to be accumulated. It could therefore be informative to see how current 3D structure prediction methods would perform when confronted with such noncompliant pieces of the RNA puzzle (Miao et al. 2015).

Efforts to fold these tetranucleotide sequences by molecular dynamics simulations are currently only partially successful, although significant progress has been made in that direction (Kührova et al. 2013; Haldar et al. 2015; Miner et al. 2016). Such modeling attempts have now to face new challenges: finding not only one, but two or more folds, while grasping their relationship with the environment. Recently, some simple procedures based on diffusion maps and Markov models found the alternative Z-turn fold of a GAAA loop (Bottaro et al. 2016). Such methods are however currently limited to small fragments—4 nt and no closing base pair in that instance. Although this represents an essential first step in assessing folding pathways, it will certainly be much more challenging to predict the occurrence of such folds or turns embedded in the core of complex RNP particles like ribosomes.

Tetraloop fold variability probably only makes for the tip of the iceberg in the folding adaptability that characterizes regulatory RNAs. Regardless of how daunting they may seem, scenarios of folding plasticity at the local level are both attractive and relevant for molecules that comprise several thousands of nucleotides and that are thought to be mostly devoid of well-defined 3D structures (Gardini and Shiekhattar 2015; Rivas et al. 2017). We could envision how this plasticity of the most basic RNA folds would be well suited to regulatory RNAs that are obligatory opportunists, by *nature*. The race is on toward “overturning more rules” about RNA structure and folding (Cech and Steitz 2014).

## MATERIALS AND METHODS

We searched the PDB (October 2016; X-ray data; resolution ≤3.0 Å) for tetranucleotide sequences in RNA hairpins that involve a 1–4 nucleobase–nucleotide interaction and an oxygen-π contact as defined below. For that purpose, we used the DSSR program (Lu et al. 2015). DSSR was also used to isolate tetranucleotide sequences embedded in loops comprising not more than eight residues. For characterizing 1–3 and 3–4 oxygen–π contacts, we specified in DSSR a 3.5 Å cutoff between the OP/O4′ oxygen atom and the nucleobase plane. In addition, the projection of the OP/O4′ oxygen on the base plane had to lie within the surface of the nucleobase aromatic cycles. A polygon-offset of 0.5 Å was used to take into account crystallographic inaccuracies. We also specified an interbase angle ≤45° to discard severely distorted 1–4 bp. Finally, we specified that no atom belonging to the tetranucleotide sequence should have a B-factor above 79 Å^2^. We visualized most of the structures, with a focus on those that appeared as borderline. In the insets of Figure 1A,C, the *d*(OP/O4′…π) histograms were calculated based on all oxygen–π contacts identified in RNA structures from the PDB and, therefore, not only on those found in tetraloop folds. To check for tetranucleotides with 1–4 interactions in NMR structures, we used the RNA FRABASE 2.0 database (Popenda et al. 2010).

For Table 1, we specified a redundancy criteria based on sequence and structural parameters (D'Ascenzo et al. 2016). If residues from two different tetranucleotide sequences (including the residues before and after the sequence) shared the same residue numbers, chain codes, ribose puckers, backbone dihedral angle sequences (we used the g+, g−, t categorization) and *syn/anti* conformations, they were considered as similar and the one with the best resolution was labeled as nonredundant. In cases of matching resolutions, the nucleotide sequence with the lowest average B-factor was selected. Alike, if in a same structure two sequences shared the same residue numbers (with different chain codes) as well as ribose puckers, backbone dihedral angle sequences, and *syn/anti* conformations, they were considered as similar and the one corresponding to the first biological unit was marked as nonredundant. To further limit redundancy in the largest ribosomal structures, we restricted our analysis to a single biological assembly. For more details, see Leonarski et al. (2016). Note that it is impossible to eliminate redundancy from such a complex structural ensemble without eliminating at the same time significant data. Here, we provide an upper limit for a truly “nonredundant” tetranucleotide fold set.

## References

[DASCENZORNA059097C1] Allain FHT, Varani G. 1995 Structure of the P1 helix from group I self-splicing introns. J Mol Biol 250: 333–353. 7608979 10.1006/jmbi.1995.0381

[DASCENZORNA059097C2] Amunts A, Brown A, Toots J, Scheres SH, Ramakrishnan V. 2015 Ribosome. *The structure of the human mitochondrial ribosome*. Science 348: 95–98. 25838379 10.1126/science.aaa1193 PMC4501431

[DASCENZORNA059097C3] Auffinger P, Westhof E. 2001 An extended structural signature for the tRNA anticodon loop. RNA 7: 334–341. 11333014 10.1017/s1355838201002382 PMC1370090

[DASCENZORNA059097C4] Batey RT. 2015 Riboswitches: still a lot of undiscovered country. RNA 21: 560–563. 25780138 10.1261/rna.050765.115 PMC4371280

[DASCENZORNA059097C5] Bottaro S, Gil-Ley A, Bussi G. 2016 RNA folding pathways in stop motion. Nucleic Acids Res 44: 5883–5891. 27091499 10.1093/nar/gkw239 PMC4937309

[DASCENZORNA059097C6] Butcher SE, Pyle AM. 2011 The molecular interactions that stabilize RNA tertiary structure: RNA motifs, patterns, and networks. Acc Chem Res 44: 1302–1311. 21899297 10.1021/ar200098t

[DASCENZORNA059097C7] Butcher SE, Dieckmann T, Feigon J. 1997 Solution structure of the conserved 16S-like ribosomal RNA UGAA tetraloop. J Mol Biol 268: 348–358. 9159475 10.1006/jmbi.1997.0964

[DASCENZORNA059097C8] Cantara WA, Bilbille Y, Kim J, Kaiser R, Leszczynska G, Malkiewicz A, Agris PF. 2012 Modifications modulate anticodon loop dynamics and codon recognition of *E. coli* tRNA^Arg1,2^. J Mol Biol 416: 579–597. 22240457 10.1016/j.jmb.2011.12.054

[DASCENZORNA059097C9] Cech TR, Steitz JA. 2014 The noncoding RNA revolution—trashing old rules to forge new ones. Cell 157: 77–94. 24679528 10.1016/j.cell.2014.03.008

[DASCENZORNA059097C10] Cheong C, Varani G, Tinoco I. 1990 Solution structure of an unusually stable RNA hairpin, 5′GGAC(UUCG)GUCC. Nature 346: 680–681. 1696688 10.1038/346680a0

[DASCENZORNA059097C11] Cheong H, Kim N, Cheong C. 2015 RNA structure: tetraloops. In ELS. Wiley, Chichester, UK.

[DASCENZORNA059097C12] Clabbers MTB, Olsthoorn RCL, Gultyaev AP. 2014 Tospovirus ambisense genomic RNA segments use almost complete repertoire of stable tetraloops in the intergenic region. Bioinformatics 30: 1800–1804. 24590440 10.1093/bioinformatics/btu122

[DASCENZORNA059097C13] Correll CC, Swinger K. 2003 Common and distinctive features of GNRA tetraloops based on a GUAA tetraloop structure at 1.4 Å resolution. RNA 9: 355–363. 12592009 10.1261/rna.2147803 PMC1370402

[DASCENZORNA059097C14] Cruz JA, Westhof E. 2009 The dynamic landscapes of RNA architecture. Cell 136: 604–609. 19239882 10.1016/j.cell.2009.02.003

[DASCENZORNA059097C15] Czudnochowski N, Ashley GW, Santi DV, Alian A, Finer-Moore J, Stroud RM. 2014 The mechanism of pseudouridine synthases from a covalent complex with RNA, and alternate specificity for U2605 versus U2604 between close homologs. Nucleic Acids Res 42: 2037–2048. 24214967 10.1093/nar/gkt1050 PMC3919597

[DASCENZORNA059097C16] D'Ascenzo L, Leonarski F, Vicens Q, Auffinger P. 2016 ‘Z-DNA like’ fragments in RNA: a recurring structural motif with implications for folding, RNA/protein recognition and immune response. Nucleic Acids Res 44: 5944–5956. 27151194 10.1093/nar/gkw388 PMC4937326

[DASCENZORNA059097C17] Delagoutte B, Moras D, Cavarelli J. 2000 Transfer-RNA aminoacylation by arginyl-transfer-RNA synthetase: induced conformations during substrates binding. EMBO J 19: 5599–5610. 11060012 10.1093/emboj/19.21.5599 PMC305789

[DASCENZORNA059097C18] Du Z, Yu J, Andino R, James TL. 2003 Extending the family of UNCG-like tetraloop motifs: NMR structure of a CACG tetraloop from coxsackievirus B3. Biochemistry 42: 4373–4383. 12693932 10.1021/bi027314e

[DASCENZORNA059097C19] Egli M, Sarkhel S. 2007 Lone pair-aromatic interactions: to stabilize or not to stabilize. Acc Chem Res 40: 197–205. 17370991 10.1021/ar068174u

[DASCENZORNA059097C20] Ennifar E, Nikulin A, Tishchenko S, Serganov A, Nevskaya N, Garber M, Ehresmann B, Ehresmann C, Nikonov S, Dumas P. 2000 The crystal structure of UUCG tetraloop. J Mol Biol 304: 35–42. 11071808 10.1006/jmbi.2000.4204

[DASCENZORNA059097C21] Fiore JL, Nesbitt DJ. 2013 An RNA folding motif: GNRA tetraloop-receptor interactions. Q Rev Biophys 46: 223–264. 23915736 10.1017/S0033583513000048

[DASCENZORNA059097C22] Gardini A, Shiekhattar R. 2015 The many faces of long noncoding RNAs. FEBS J 282: 1647–1657. 25303371 10.1111/febs.13101 PMC4520312

[DASCENZORNA059097C23] Garst AD, Edwards AL, Batey RT. 2011 Riboswitches: structures and mechanisms. Cold Spring Harb Perspect Biol 3: a003533. 20943759 10.1101/cshperspect.a003533 PMC3098680

[DASCENZORNA059097C24] Gottstein-Schmidtke SR, Duchardt-Ferner E, Groher F, Weigand JE, Gottstein D, Suess B, Wohnert J. 2014 Building a stable RNA U-turn with a protonated cytidine. RNA 20: 1163–1172. 24951555 10.1261/rna.043083.113 PMC4105743

[DASCENZORNA059097C25] Grosjean H, Westhof E. 2016 An integrated, structure- and energy-based view of the genetic code. Nucleic Acids Res 44: 8020–8040. 27448410 10.1093/nar/gkw608 PMC5041475

[DASCENZORNA059097C26] Gutell RR, Cannone JJ, Konings D, Gautheret D. 2000 Predicting U-turns in ribosomal RNA with comparative sequence analysis. J Mol Biol 300: 791–803. 10891269 10.1006/jmbi.2000.3900

[DASCENZORNA059097C27] Haldar S, Kuhrova P, Banas P, Spiwok V, Sponer J, Hobza P, Otyepka M. 2015 Insights into stability and folding of GNRA and UNCG tetraloops revealed by microsecond molecular dynamics and well-tempered metadynamics. J Chem Theory Comput 11: 3866–3877. 26574468 10.1021/acs.jctc.5b00010

[DASCENZORNA059097C28] Hall KB. 2015 Mighty tiny. RNA 21: 630–631. 25780168 10.1261/rna.050567.115 PMC4371310

[DASCENZORNA059097C29] Hendrix DK, Brenner SE, Holbrook SR. 2005 RNA structural motifs: building blocks of a modular biomolecule. Q Rev Biophys 38: 221–243. 16817983 10.1017/S0033583506004215

[DASCENZORNA059097C30] Heus HA, Pardi A. 1991 Structural features that give rise to the unusual stability of RNA hairpins containing GNRA loops. Science 253: 191–194. 1712983 10.1126/science.1712983

[DASCENZORNA059097C31] Hsiao C, Mohan S, Hershkovitz E, Tannenbaum A, Williams LD. 2006 Single nucleotide RNA choreography. Nucleic Acids Res 34: 1481–1491. 16531589 10.1093/nar/gkj500 PMC1401506

[DASCENZORNA059097C32] Ihle Y, Ohlenschlager O, Hafner S, Duchardt E, Zacharias M, Seitz S, Zell R, Ramachandran R, Gorlach M. 2005 A novel cGUUAg tetraloop structure with a conserved yYNMGg-type backbone conformation from cloverleaf 1 of bovine enterovirus 1 RNA. Nucleic Acids Res 33: 2003–2011. 15814817 10.1093/nar/gki501 PMC1074726

[DASCENZORNA059097C33] Jucker FM, Pardi A. 1995a GNRA tetraloops make a U-turn. RNA 1: 219–222. 7585251 PMC1369075

[DASCENZORNA059097C34] Jucker FM, Pardi A. 1995b Solution structure of the CUUG hairpin loop: a novel RNA tetraloop motif. Biochemistry 34: 14416–14427. 7578046 10.1021/bi00044a019

[DASCENZORNA059097C35] Jucker FM, Heus HA, Yop PF, Moors HHM, Pardi A. 1996 A network of heterogeneous hydrogen bonds in GNRA tetraloops. J Mol Biol 264: 968–980. 9000624 10.1006/jmbi.1996.0690

[DASCENZORNA059097C36] Keating KS, Toor N, Pyle AM. 2008 The GANC tetraloop: a novel motif in the group IIC intron structure. J Mol Biol 383: 475–481. 18773908 10.1016/j.jmb.2008.08.043 PMC2574657

[DASCENZORNA059097C37] Kim SH, Sussman JL. 1976 π-turn is a conformational pattern in RNA loops and bends. Nature 260: 645–646. 772447 10.1038/260645a0

[DASCENZORNA059097C38] Klosterman PS, Hendrix DK, Tamura M, Holbrook SR, Brenner SE. 2004 Three-dimensional motifs from the SCOR, structural classification of RNA database: extruded strands, base triples, tetraloops and U-turns. Nucleic Acids Res 32: 2342–2352. 15121895 10.1093/nar/gkh537 PMC419439

[DASCENZORNA059097C39] Konings DAM, Gutell R. 1995 A comparison of thermodynamic foldings with comparatively derived structures of 16S and 16S like rRNAs. RNA 1: 559–574. 7489516 PMC1369301

[DASCENZORNA059097C40] Kührova P, Banas P, Best RB, Sponer J, Otyepka M. 2013 Computer folding of RNA tetraloops? Are we there yet? *J.* Chem Theory Comput 9: 2115–2125. 10.1021/ct301086z 26583558

[DASCENZORNA059097C41] Lee CW, Li L, Giedroc DP. 2011 The solution structure of coronaviral stem–loop 2 (SL2) reveals a canonical CUYG tetraloop fold. FEBS Lett 585: 1049–1053. 21382373 10.1016/j.febslet.2011.03.002 PMC3086565

[DASCENZORNA059097C42] Leonarski F, D'Ascenzo L, Auffinger P. 2016 Mg^2+^ ions: do they bind to nucleobase nitrogens? Nucleic Acids Res. 10.1093/nar/gkw1175. PMC5314772 27923930

[DASCENZORNA059097C43] Leontis NB, Westhof E. 2001 Geometric nomenclature and classification of RNA base pairs. RNA 7: 499–512. 11345429 10.1017/s1355838201002515 PMC1370104

[DASCENZORNA059097C44] Liu PH, Li LC, Keane SC, Yang D, Leibowitz JL, Giedroc DP. 2009 Mouse hepatitis virus stem–loop 2 adopts a uYNMG(U)a-like tetraloop structure that is highly functionally tolerant of base substitutions. J Virol 83: 12084–12093. 19759148 10.1128/JVI.00915-09 PMC2786756

[DASCENZORNA059097C45] Lu XJ, Bussemaker HJ, Olson WK. 2015 DSSR: an integrated software tool for dissecting the spatial structure of RNA. Nucleic Acids Res 43: e142. 26184874 10.1093/nar/gkv716 PMC4666379

[DASCENZORNA059097C46] Melchers WJG, Zoll J, Tessari M, Bakhmutov DV, Gmyl AP, Agol VI, Heus HA. 2006 A GCUA tetranucleotide loop found in the poliovirus *ori*L by in vivo SELEX (un)expectedly forms a YNMG-like structure: extending the YNMG family with GYYA. RNA 12: 1671–1682. 16894217 10.1261/rna.113106 PMC1557697

[DASCENZORNA059097C47] Miao Z, Adamiak RW, Blanchet MF, Boniecki M, Bujnicki JM, Chen SJ, Cheng C, Chojnowski G, Chou FC, Cordero P, 2015 RNA-puzzles round II: assessment of RNA structure prediction programs applied to three large RNA structures. RNA 21: 1066–1084. 25883046 10.1261/rna.049502.114 PMC4436661

[DASCENZORNA059097C48] Miner JC, Chen AA, Garcia AE. 2016 Free-energy landscape of a hyperstable RNA tetraloop. Proc Natl Acad Sci 113: 6665–6670. 27233937 10.1073/pnas.1603154113 PMC4914146

[DASCENZORNA059097C49] Nasalean L, Stombaugh J, Zirbel CL, Leontis NB. 2009 RNA 3D structural motifs: definition, identification, annotation, and database searching. In Non-protein coding RNAs (ed. Walter NG, et al.), pp. 1–26. Springer, Berlin, Heidelberg.

[DASCENZORNA059097C50] Nozinovic S, Furtig B, Jonker HR, Richter C, Schwalbe H. 2010 High-resolution NMR structure of an RNA model system: the 14-mer cUUCGg tetraloop hairpin RNA. Nucleic Acids Res 38: 683–694. 19906714 10.1093/nar/gkp956 PMC2811024

[DASCENZORNA059097C51] Oberstrass FC, Lee A, Stefl R, Janis M, Chanfreau G, Allain FH. 2006 Shape-specific recognition in the structure of the Vts1p SAM domain with RNA. Nat Struct Mol Biol 13: 160–167. 16429156 10.1038/nsmb1038

[DASCENZORNA059097C52] Popenda M, Szachniuk M, Blazewicz M, Wasik S, Burke EK, Blazewicz J, Adamiak RW. 2010 RNA FRABASE 2.0: an advanced web-accessible database with the capacity to search the three-dimensional fragments within RNA structures. BMC Bioinformatics 11: 231. 20459631 10.1186/1471-2105-11-231 PMC2873543

[DASCENZORNA059097C53] Proctor DJ, Schaak JE, Bevilacqua JM, Falzone CJ, Bevilacqua PC. 2002 Isolation and characterization of a family of stable RNA tetraloops with the motif YNMG that participate in tertiary interactions. Biochemistry 41: 12062–12075. 12356306 10.1021/bi026201s

[DASCENZORNA059097C54] Prostova MA, Gmyl AP, Bakhmutov DV, Shishova AA, Khitrina EV, Kolesnikova MS, Serebryakova MV, Isaeva OV, Agol VI. 2015 Mutational robustness and resilience of a replicative *cis*-element of RNA virus: promiscuity, limitations, relevance. RNA Biol 12: 1338–1354. 26488412 10.1080/15476286.2015.1100794 PMC4829312

[DASCENZORNA059097C55] Quigley GJ, Rich A. 1976 Structural domains of transfer RNA molecules. Science 194: 796–806. 790568 10.1126/science.790568

[DASCENZORNA059097C56] Rivas E, Clements J, Eddy SR. 2017 A statistical test for conserved RNA structure shows lack of evidence for structure in lncRNAs. Nat Methods 14: 45–48. 27819659 10.1038/nmeth.4066 PMC5554622

[DASCENZORNA059097C57] Sahu B, Khade PK, Joseph S. 2012 Functional replacement of two highly conserved tetraloops in the bacterial ribosome. Biochemistry 51: 7618–7626. 22938718 10.1021/bi300930r PMC3786584

[DASCENZORNA059097C58] Schultes EA, Bartel DP. 2000 One sequence, two ribozymes: implications for the emergence of new ribozyme folds. Science 289: 448–452. 10903205 10.1126/science.289.5478.448

[DASCENZORNA059097C59] Schwalbe M, Ohlenschlager O, Marchanka A, Ramachandran R, Hafner S, Heise T, Gorlach M. 2008 Solution structure of stem–loop α of the hepatitis B virus post-transcriptional regulatory element. Nucleic Acids Res 36: 1681–1689. 18263618 10.1093/nar/gkn006 PMC2275152

[DASCENZORNA059097C60] Sundaram M, Durant PC, Davis DR. 2000 Hypermodified nucleosides in the anticodon of tRNA^Lys^ stabilize a canonical U-turn structure. Biochemistry 39: 12575–12584. 11027137 10.1021/bi0014655

[DASCENZORNA059097C61] Tanaka T, Furuta H, Ikawa Y. 2013 Natural selection and structural polymorphism of RNA 3D structures involving GNRA loops and their receptor motifs. In RNA nanotechnology and therapeutics (ed. Guo P), pp. 109–120. CRC Press, FL.

[DASCENZORNA059097C62] Theimer CA, Finger LD, Feigon J. 2003 YNMG tetraloop formation by a dyskeratosis congenita mutation in human telomerase RNA. RNA 9: 1446–1455. 14624001 10.1261/rna.5152303 PMC1370499

[DASCENZORNA059097C63] Woese CR, Winker S, Gutell RR. 1990 Architecture of ribosomal RNA: constraints on the sequence of “tetra-loops.” Proc Natl Acad Sci 87: 8467–8471. 2236056 10.1073/pnas.87.21.8467 PMC54977

[DASCENZORNA059097C64] Woodson SA. 2015 RNA folding retrospective: lessons from ribozymes big and small. RNA 21: 502–503. 25780113 10.1261/rna.051110.115 PMC4371255

[DASCENZORNA059097C65] Zhao Q, Huang HC, Nagaswamy U, Xia Y, Gao X, Fox GE. 2012 UNAC tetraloops: to what extent do they mimic GNRA tetraloops? Biopolymers 97: 617–628. 22605553 10.1002/bip.22049

[DASCENZORNA059097C66] Zirbel CL, Sponer JE, Sponer J, Stombaugh J, Leontis NB. 2009 Classification and energetics of the base-phosphate interactions in RNA. Nucleic Acids Res 37: 4898–4918. 19528080 10.1093/nar/gkp468 PMC2731888

[DASCENZORNA059097C67] Zoll J, Hahn MM, Gielen P, Heus HA, Melchers WJ, van Kuppeveld FJ. 2011 Unusual loop-sequence flexibility of the proximal RNA replication element in EMCV. PLoS One 6: e24818. 21935472 10.1371/journal.pone.0024818 PMC3173479

